# Newly acquired word-action associations trigger auditory cortex activation during movement preparation: Implications for Hebbian plasticity in action word learning

**DOI:** 10.1371/journal.pone.0325977

**Published:** 2025-07-02

**Authors:** Vera Tretyakova, Anna Pavlova, Vasily Arapov, Anna Rytikova, Alicia Vorobiova, Andrey Prokofyev, Boris Chernyshev, Tatiana Stroganova

**Affiliations:** 1 Center for Neurocognitive Research (MEG Center), Moscow State University of Psychology and Education, Moscow, Russia; 2 School of Psychology, HSE University, Moscow, Russia; 3 Department of Higher Nervous Activity, Lomonosov Moscow State University, Moscow, Russia; Max-Planck-Institut fur Kognitions- und Neurowissenschaften, GERMANY

## Abstract

Action word learning is believed to rely on mechanisms of Hebbian learning. However, this biological mechanism requires activation of the neural assemblies representing a word form and a corresponding movement to repeatedly overlap in time. In reality, though, these associated events could be separated by seconds. In the current MEG study, we examined trial-and-error learning of associations between novel auditory pseudowords and movements of specific body parts. We aimed to explore how the brain bridges the temporal gap between the transient activity evoked by auditory input and the preparatory motor activation before the corresponding movement. To address this, we compared learning-induced changes in neuromagnetic responses locked to the onset of the stimulus and to the onset of the movement. As learning progressed, both types of neural responses showed sustained enhancement during the delay period between the auditory pseudoword and the required movement. Cortical sources of this learning-induced increase were localized bilaterally in the lateral and medial temporal cortices. Notably, the learning effect was significantly stronger when measured time-locked to the movement onset, rather than to the pseudoword onset. This suggests that once pseudoword-movement associations were reliably acquired, extensive regions of the auditory cortex were reactivated in synchrony with the preparation for the upcoming movement. Such reactivation likely served to bring together in time the representations of the correct action and the preceding auditory cue. This temporal alignment could enable Hebbian learning, leading to long-lasting synaptic changes in temporally correlated neural assemblies.

## Introduction

The embodied theoretical perspective suggests that cognition is rooted in the brain action, perception, and emotion systems [1–3]. In recent decades, this view has gained increasing influence, particularly in language research [4,5]. Within the embodied framework, language acquisition is considered to rely on general – Hebbian – mechanisms of associative learning [6–8], i.e., activity-dependent modification of synaptic strengths triggered by repeated co-activation of two neuronal representations [9,10]. For example, according to this view, meaning of action words is acquired because of repeated simultaneous activation of neural assemblies coding the auditory representation of the word and neurons programming the corresponding physical movement. Such co-activation eventually leads to strengthened synaptic connections and a formation of distributed and interrelated “cell assemblies” [9]. As a result of these plastic changes, the auditory signal presented alone begins to activate both neuronal populations – not only the auditory areas, but the motor regions as well.

Repeated co-activation of acoustic and motor representations strengthens the reciprocal link between sound and action, mirroring the articulatory–auditory loop central to both speech production and comprehension [11]. In self-produced speech, this dynamic sensorimotor interplay mainly relies on reciprocal fibers of the frontotemporal arcuate fasciculus, which directly connect the auditory cortex with motor speech regions in the frontal cortex [12] mediating their joint activation [11,13,14]. In contrast, mapping heard spoken words onto corresponding body movements is thought to engage multimodal regions, including the supplementary motor area, prefrontal cortex, anterior temporal lobe, and posterior parietal cortex [8,15,16]. Through their extensive connectivity, these regions indirectly integrate motor and auditory representations across primary, secondary, and multimodal areas.

Indirect evidence for the applicability of the Hebbian learning principle to human language was mainly derived from the neuroimaging findings on joint activation in both auditory and somatotopically organized motor cortical areas driven by auditory presentations of well-learned action words [17]. Several fMRI studies observed that passive listening to face, hand, and leg-related action verbs (e.g., “lick,” “pick,” or “kick”) activated the left primary motor and/or premotor cortex in, crucially, roughly somatotopic manner [18–20]. Time-sensitive techniques such as MEG and TMS [17,21–23] showed that the language-induced motor activation occurred at the very early stage of words processing (earlier than ~200 ms) indicating that the motor activation is triggered automatically by a word presentation and does not reflect slow post-semantic processes. Together, these findings were interpreted as evidence of the existence of robust connections between acoustic representations of action words and corresponding motor representations. The possibility of formation of such connections via Hebbian learning was further supported by computational modeling of action word learning [15,24].

Biologically-based concepts of action word learning have an important assumption that, throughout the learning procedure, the auditory activation produced by an action word and programming of corresponding motor commands in the motor cortex should overlap in time or follow each other with a very small delay [25]. Particularly, studies of spike timing dependent plasticity (STDP) – a biological mechanism which is often identified with Hebbian learning – have defined that time windows between pre- and post-synaptic inputs could not exceed few tens of milliseconds to STDP takes place [26–29]. However, in a natural environment such learning can occur despite a temporal gap between an action and an associated word on a timescale of hundreds or even thousands of milliseconds. To explain action word learning by STDP, one must account for this large discrepancy in timescales, and find a mechanism that bridges up this gap in time.

Available neuronal studies on animals have suggested two possible mechanisms that could allow learning to occur in the adult brain, despite the lack of precise temporal coincidence between the sensory signal and the motor response. One approach to this problem is to assume that along the course of associative learning, novel action words start to generate persistent auditory responses that outlast these stimuli long enough to overlap with the associated motor command. Since Hebb’s idea [9] that was put forward more than 50 years ago, mnemonic activity has been hypothesized to be sustained by synaptic reverberation in a recurrent circuit [30,31]. We will address such presumable prolongation of activity triggered by stimulus onset as a “retrospective” mechanism. This possibility was supported by numerous findings of increased neural activity during the delay period in working memory tasks in monkeys [32] and humans [33].

Another option is that the input representation could be “prospectively” reactivated shortly before the movement onset so that the two populations of auditory and motor neurons would be activated concurrently. This view is in line with proposals that stimulus-related activity can subside during the delay and then reappear when a task requires [34,35]. Similarly, in an associative learning task where participants were acquiring associations between short videos and unrelated nouns, “reappearance” of the video-specific neural response in EEG signal was shown during delayed presentation of the noun associate [36]. However, the authors did not test directly whether the video-specific pattern reflected maintenance of the stimulus-related activity over the delay period or it was ignited anew by the noun presentation.

Whereas the two models are not mutually exclusive – and both mechanisms can coexist and provide cumulative effects – the critical difference between them is timing of the auditory activation during the delay period between presentation of a novel action word and a chosen motor response. The “retrospective” model predicts that, over the course of learning, a rapidly decaying auditory neural response will be replaced by the more persistent auditory activation. Such prolonged auditory activation, although decreasing over time, will still last until the end of the delay period. In contrast, if the co-occurrence of auditory and motor activation during the delay period is driven by “prospective” reactivation of the former representation, its neural manifestations in the form of increased auditory activation will be strongest immediately before the motor act.

Our recent magnetoencephalography (MEG) study in human adults indicated that passive listening to pseudowords previously associated with specific motor actions through active operant conditioning evokes differential brain activation in auditory and other speech areas of the left hemisphere compared with pseudowords without motor associations [37]. The emergent difference in neural responsiveness that persisted throughout the passive listening of an auditory cue proved that active learning of auditory-action association drives rapid cortical plasticity in auditory cortex of human adults, and that finding was a necessary prerequisite for the current study.

In the current study, we investigated the learning-induced neural changes during active formation of pseudoword-action association in the same subjects. We applied a set of eight tightly controlled action-related and non-action-related pseudowords, whose relatedness to action of different body parts was gradually established by the participants through trial-and-error learning.

First, we aimed to investigate if the temporal gap between an auditory pseudoword and a motor action is indeed filled with the persistent activation of auditory neural representations. For this purpose, we compared brain activity in response to the auditory pseudowords between early and advanced stages of learning. We expected that, as the participants shifted from random guessing to purposeful responding according to an emerging understanding of the associative rules, a neural response to the auditory pseudowords would become strengthened and prolonged to ensure co-occurrence and, thus, association of the auditory activation with the preparation of the chosen motor program. We further anticipated a more pronounced learning-related enhancement for newly learned “action” pseudowords compared to non-action-related ones, assuming this effect reflects auditory-motor integration rather than mere pseudoword memorization. If, however, learning effects were primarily driven by memory retrieval, similar increases in neural responses would be expected for both action and non-action pseudowords.

If this persistent activation was present during the delay period, we aimed to probe which of the two suggested neural accounts – “retrospective” or “prospective” – better explains this result. We planned to compare cortical responses time-locked to the auditory stimulus onset and to the motor response onset. If the “retrospective” hypothesis is correct, we could expect a learning-induced prolongation of auditory cue-evoked activation which would be the most evident in the stimulus-locked data. Specifically, this effect may enhance the descending slope within the temporal window of the N400 component of the pseudoword-evoked neural response, which is widely recognized as a neural marker of information retrieval from lexical-semantic memory [38–41].

The “prospective” hypothesis predicts that the advanced learning stage would be mainly characterized by the appearance of the auditory activation that, paradoxically, would be largely driven by preparation for motor action. Hence, the auditory activation would be time-locked to the onset of the motor response and would co-occur with activation of the premotor areas that form a central node of action preparation.

## Materials and methods

### Participants

Twenty-eight adult native Russian speakers (mean age 25.5 years, range 19–38 years, 17 males) were recruited to participate in the study during the period from December 1, 2021 to December 31, 2022. All participants were right-handed (Edinburgh Handedness Inventory [42]); they had normal hearing and reported no history of neurological or psychiatric disorders. Nine participants did not have a sufficient number of trials in one of the conditions (see “Epoch selection and MEG data preprocessing” section for details) and we excluded them from further analyses. The final sample comprised 19 participants. The study was conducted following the ethical principles regarding human experimentation (Helsinki Declaration) and was approved by the Ethics Committee of the Moscow State University of Psychology and Education as reflected in the protocol of the meeting of the Ethics Committee No.6 dated November 30, 2021. A written informed consent was obtained from all participants prior to the experiment.

### Stimuli and procedure

The auditory stimuli (pseudowords) were designed so that their acoustic and phonetic properties were controlled and balanced while their lexical status was manipulated by learning (for their detailed description see [37]). We used nine consonant-vowel (CV) syllables, which were organized in eight disyllabic (C_1_V_1_ C_2_V_2_) novel meaningless word-forms. The resulting pseudowords complied with Russian language phonetics and phonotactic constraints. During the associative learning procedure, four of these word stimuli were associated with a unique action performed by one of four body extremities (action-related pseudowords, APW), while the other four were not assigned any motor response (non-action-related pseudowords, NPW).

The first two phonemes (C_1_V_1_) formed the syllable “hi” (xʲˈi) that was identical for all pseudowords used. The next two phonemes (C_2_ and V_2_) were balanced across APW and NPW stimuli in such a way to ensure that acoustic and phonetic features were fully matched between the two stimuli types (Table 1). The third phonemes (C2) – one of the four consonants “ch” (t͡ɕ), “sh” (ʂ), “s” (s̪), “v” (v) – occurred in both APW and NPW stimuli and signaled which extremity a subject should be prepared to use (right hand, left hand, right foot, or left foot). The fourth phonemes (V_2_: vowel “a” (ə) or “u” (ʊ)) were counterbalanced between APW and NPW conditions being included in the two stimuli of each type so that to form eight unique phonemic combinations. Thus, only the fourth phoneme allows recognition of all pseudowords used in the experiment. Onset of the fourth phoneme henceforth will be referred to as “word-form uniqueness point”.

**Table 1:** Stimulus-to-response mapping.

Action-related pseudowords			Non-action-related pseudowords		
Pseudoword	Pronunciation	Assigned movement	Pseudoword	Pronunciation	Assigned movement
hivu	[xʲˈivʊ}	right hand	hiva	[xʲˈivə}	none
hicha	[xʲˈit͡ɕə}	left hand	hichu	[xʲˈit͡ɕʊ}	none
hisa	[xʲˈis̪ə}	right foot	hisu	[xʲˈis̪ʊ}	none
hishu	[xʲˈiʂʊ}	left foot	hisha	[xʲˈiʂə}	none

As can be seen in Table 1, the phonetic composition of the stimuli and stimulus-to-response mapping complied with a full within-subject counterbalanced design, in relation to the third and fourth phonemes, as well as in relation to movements by left/right and upper/lower extremities.

All stimuli were digitally recorded (PCM, 32 bit, 22050 Hz, 1 channel, 352 kbps) by a female native Russian speaker’s voice in a sound-attenuated booth. We generated eight pseudoword stimuli by way of cross-splicing the sound recordings. We used a single variant of the first syllable (C_1_V_1_), four variants of the third phoneme (C_2_) and two variants of the last vowel (V_2_). All pseudowords were pronounced with the first vowel “I” stressed. The amplitude of the sound recordings was digitally equalized by maximal power, which corresponded to the stressed vowel “I”. Cross-splicing and normalization of the recorded stimuli was done using Adobe Audition CS6.5 software. The mean (± std) duration of the pseudowords was approximately 510 (± 25 ms), with word-form uniqueness point occurring approximately 360 (± 11 ms) after the stimulus onset.

Two additional non-speech sounds were used as positive and negative feedback signals, each 400 ms in length. The positive and negative stimuli differed in their spectral frequency (ranges were approximately 400–800 Hz for positive and 65–100 Hz for negative feedback), with spectral maxima increasing in frequency over time for the positive feedback and decreasing for the negative feedback.

Hand responses were recorded using hand-held buttons (package 932, CurrentDesigns, Philadelphia, PA, USA) pressed by the right or left thumb while foot movements were recorded by means of custom-made pedals pushed by the right or left foot. In order to minimize movement artifacts, we kept the trajectory of the movements rather short (<1 cm for buttons and <3 cm for pedals). When pushed, buttons and pedals interrupted a laser light beam delivered via fiber optic cable. The responses recorded from pedals and buttons were automatically labeled as “correct” or “erroneous” after each trial in accordance with the task rules (see below).

For the entire length of the experiment, the participants were comfortably seated in the MEG apparatus inside of an electromagnetically and acoustically shielded room (see below). Pseudowords were presented binaurally via plastic ear tubes in an interleaved quasi-random order, at 60 dB SPL. The experiment was implemented using the Presentation 14.4 software (Neurobehavioral systems, Inc., Albany, CA, USA).

**Fig 1:**
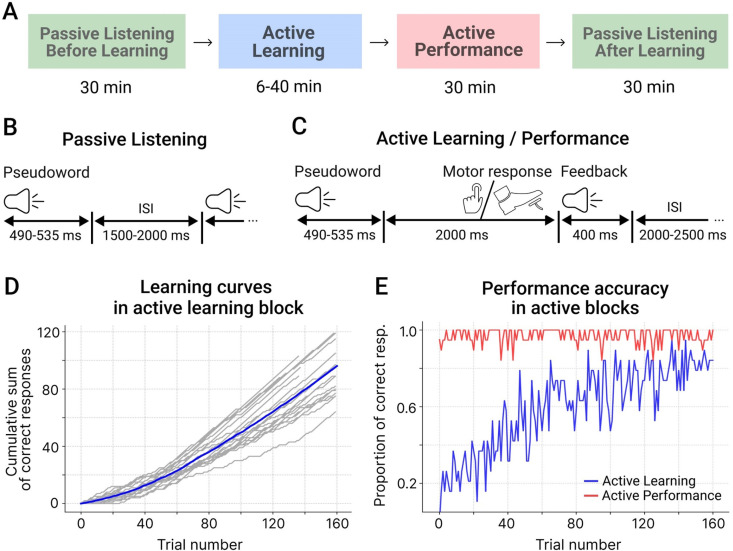
Experimental procedure and behavioral results. A. Sequence and approximate duration of experimental blocks. B. Trial structure in the passive listening block. C. Trial structure in the active learning and active performance blocks. D. Cumulative performance accuracy over the first 160 trials of the active learning block. The gray lines represent individual learning curves, the blue line – the grand average. E. The proportion of correct responses over the first 160 trials of the active learning (blue) and active performance (red).

The experiment comprised four blocks presented consequently in a fixed order for all participants: (1) passive listening before learning, (2) active learning, (3) active performance, and (4) passive listening after learning (Fig 1A). The whole experiment lasted approximately 2 hours. Here, we analyzed the data from two active blocks (results for the passive blocks were reported in [37]).

During two passive listening blocks the participants were auditorily presented with the pseudowords in a pseudorandom interleaved order. An average interstimulus interval (ISI) was 1750 ms with a random jitter between 1500 and 2000 ms at one-millisecond steps (Fig 1B). Each passive block consisted of 400 stimuli (each of eight pseudowords was presented 50 times) and lasted around 30 min. To prevent the participants from paying attention to the auditory stimuli, they were presented with a silent movie of their choice projected on the screen positioned at eye-level.

Upon completion of the first passive block, the participants were instructed that during the following active blocks they had to deduce the unique associations between each of the presented eight pseudowords and movements of their own body parts. In order to do that, they were required either to respond to each pseudoword by using one of the four body extremities or to withhold motor response, and then listened to positive and negative feedback signals informing whether the action was correct or erroneous. The instruction did not contain any other clues: the behavioral procedure implied trying a variety of auditory cue-action associations and eventually settling down on those that led to positive reinforcement, thus complying with the requirements of operant learning [43].

During the active blocks, participants were instructed to keep their gaze at the fixation cross in the center of the presentation screen, with the purpose of minimizing artifacts caused by eye movements. Each pseudoword was followed by a feedback signal. Positive feedback was given if a participant complied with the task rules, i.e., executed a correct movement to an APW stimulus or committed no response to an NPW stimulus (Table 1). Negative feedback followed three kinds of errors: (i) no response to an APW stimulus; (ii) a motor response to an APW stimulus performed with a wrong extremity; (iii) any response to an NPW stimulus. Both positive and negative feedback were presented 2000 ms after the end of the pseudoword stimulus (Fig 1C). The average inter-stimulus interval (from the end of the feedback stimulus until the onset of the next pseudoword stimulus) was 2250 ms, randomly jittered between 2000 and 2500 ms at one-millisecond steps.

In line with the operant learning procedure, the number of trials (repetitions) was not standardized across participants. Instead, the goal was to ensure that all participants reached the same performance criterion—accurately memorizing all eight pseudoword-action associations. Although this approach led to variability in the number of repetitions due to individual differences in learning rate, it ensured that neural responses reflected the successful acquisition of the association rules rather than mere exposure to a fixed number of stimuli. Accordingly, the number of stimuli presented in the active learning block varied across participants. The block ended if a participant reached the learning criterion or if 480 stimuli were presented in total, whichever came first. The learning criterion required that a participant made correct responses in at least four out of five consecutive presentations of each of the eight pseudowords. Correctness of the behavioral response as well as whether a participant met the learning criterion was automatically checked after each trial using custom-made scripts. One participant failed to reach the learning criterion and thus went through all 480 trials in the learning block. Considering that his overall hit rate during the next active performance block was well within the range of performance of the other participants, we included this participant in all analyses. There was considerable variation between the participants in the duration of the active learning block: the number of trials required for a participant to reach the learning criterion ranged from 74 (9 repetitions for each pseudoword) to 480 (60 repetitions for each pseudoword), with the duration of the learning block varied from 6 to 40 min.

In the next active performance block (Fig 1A), the participants were asked to repeat the same procedure. The only difference between the two active blocks was that the active performance block included a fixed number of 320 trials, with each of the 8 pseudowords presented 40 times, and lasted approximately 30 min.

The participants were offered short breaks between the blocks (10 min between the active performance block and the second passive block and 3 min between other blocks), during which they rested while remaining seated in the MEG apparatus.

### MEG data acquisition

The experiment was conducted at the research facility “Center for Neurocognitive Research (MEG-Center)” of MSUPE. MEG was recorded in a magnetically shielded room (AK3b, Vacuumschmelze GmbH, Hanau, Germany) using a dc-SQUID Neuromag VectorView system (Elekta-Neuromag, Helsinki, Finland), which has 306 MEG channels (204 planar gradiometers and 102 magnetometers). The MEG signals were recorded with a band-pass filter 0.1–330 Hz, digitized at 1000 Hz, and stored for offline analysis.

Participants’ head shapes were measured using a 3Space Isotrack II System (Fastrak Polhemus, Colchester, VA, USA) by digitizing three anatomical landmark points (nasion, left, and right preauricular points) and additional randomly distributed 60–100 points on the scalp. Head position and orientation were continuously monitored during MEG recording by four Head Position Indicator coils. Two pairs of electrooculographic electrodes located above and below the left eye and at the outer canthi of both eyes were used to record vertical and horizontal eye movements.

Biological artifacts and other environmental magnetic sources originating outside the head were removed from MEG data using tSSS algorithm [44] implemented in the MaxFilter program (Elekta Neuromag software). For further sensor-level analysis, MEG data were converted to a standard head position (x = 0 mm; y = 0 mm; z = 45 mm). Static bad channels were excluded from further processing. Correction of biological artifacts (caused by eye-blinks and heart-beats) was performed by means of the ICA method in a semi-automatic procedure (by means of MNE-Python software [45]). The independent components for exclusion were, first, automatically identified using EOG or ECG recordings as references and, then, inspected manually. On average, one component for eye-related and two-three components for heartbeat-related artifacts were removed. At the next step, following procedure used in previous studies [37,46,47], we identified epochs with elevated muscle activity by high-pass filtering the MEG signal above 60 Hz and computing the mean absolute amplitude at each channel. Epochs exhibiting maximal amplitudes exceeding 9 standard deviations above the across-channel average in more than 25% of channels were excluded from analysis. The remaining epochs were then visually inspected to ensure the absence of muscle artifacts. On average, 15 trials per participant were excluded in the active learning block and 23 trials per participant in the active performance block, corresponding to 5% and 7% of the total number of trials, respectively. The number of excluded epochs did not significantly differ between conditions (t(18)=−1.91, p = 0.07).

### Behavioral analysis

The participants’ performance was evaluated as (i) a mean number of correct responses for four APW pseudowords, which were associated with movements (correct movement execution), for four NPW pseudowords, which were not (correct refraining from any movement), and the total number of correct APW and NPW trials, and (ii) a mean latency of the motor responses for APW pseudowords. These two parameters were calculated separately for each of the two active blocks. The performance between two types of stimuli within each block, and between the blocks was compared using the paired t-test. All the above analyses were conducted using functions of SciPy package in Python.

To characterize the learning curve, we used the cumulative record of correct responses as a function of trials [48], which is calculated as a running sum of successive behavioral measurements. Changes in the slope of this curve correspond to changes in the level of performance (Fig 1D). Given individual variability in the learning curves, to visualize the general course of performance at the group level, we plotted a proportion of subjects, who gave a correct response at each consecutive trial, for each block separately (Fig 1E).

Considering that during the active performance block the participants reached a near-ceiling level of accuracy (Fig 1E), we resorted to a response times (RT) analysis in order to check whether learning progressed further during the advanced stage of the experiment. For this purpose, we applied linear mixed-effects models (LMEM). We used an RT in each trial in the active performance block as a dependent variable with a corresponding trial number and a limb the participants responded with as fixed factors. Differences in RT between the participants were attributed to random effects. The full model was as follows:


$$\begin{array}{c} \text{RT }\sim \text{ stim}\_\text{number*limb }+\;(1|\text{subject}) \end{array}$$

The linear mixed-effects models were built with the “lmer()” function available in the lme4 package for R [49].

### Epoch selection and MEG data preprocessing

To probe two different explanations of putative learning-induced contingency between auditory and motor activation during the time delay between a heard pseudoword and a performed motor response (or absence of a response), we evaluated both stimulus-locked and response-locked brain activity. In both analyses, we focused on the difference in MEG data between two conditions: the early and the advanced stages of auditory-motor association learning (ESL and ASL respectively), during which the participants gradually acquired the association rules between the pseudowords and the actions of body extremities (or alternatively with the absence of any actions). We adopted the following strategy for epoch selection.

For both stimulus-locked and response-locked analyses, we extracted 28 earliest trials from the active learning block (ESL condition) and 28 latest trials from the active performance block (ASL condition), and we did that extraction separately for two types of trials: with a motor response (APW) and without a motor response (NPW). We included only correct trials in the analysis, discarding any incorrect responses. Because participants responded using all four extremities, we balanced the number of trials with movements by each extremity to control for potential effects or interactions associated with specific limbs. From each active block, we extracted seven correct trials per extremity, resulting in 28 trials per condition and trial type. Nine participants, who completed the active learning block too quickly to allow extraction of a sufficient number of trials were excluded, and the following analyses were done in 19 participants.

For both sensor-level and source-level analyses, the trials were epoched from −700–2500 ms relative to pseudoword onset for the stimulus-locked data; and from −1500–1000 ms relative to the motor response onset for the response-locked data. Both stimulus-locked and response-locked event-related fields (ERFs) were baseline-corrected using 450 ms pre-stimulus window (from −500 ms to −50 ms before the stimulus onset). We selected the baseline interval duration based on Brunia and van Boxtel [50], who emphasized the importance of longer baselines (approximately 500 ms) when investigating slow ERP deflections, such as those anticipated in our study.

### Sensor-level analysis

First, we will describe the general framework of how we treated the sensor-level data in both stimulus- and response-locked analyses.

For the sensor-level analysis, MEG data were downsampled to 300 frames per second. At sensor-level, we used MEG signal from planar gradiometers which are known to be less sensitive to distant sources than magnetometers and, thus, provide clearer and easier-to-interpret picture [51–53].

To examine and illustrate general ERF dynamics over time in a specific condition, we computed root mean square (RMS) over ERFs values across all 204 gradiometers at each time point, thus obtaining time courses of global field power (GFP). The baseline values were subtracted from GFP data. The resulting GFP time courses were compared between conditions of interest (Passive Listening vs ESL, ESL vs ASL) using the paired two-tailed t-test with FDR correction [54] for multiple comparisons for the number of time points.

For topographical analyses over the whole head, we computed RMS values of ERFs separately for each pair of gradiometers (hereafter referred to as combined gradiometers, or simply sensors). The resulting RMS values were averaged within the time windows of interest (described in details below) for each subject and experimental condition separately. Significance of the between-condition differences at each sensor was assessed using the paired two-tailed t-test with FDR correction for multiple comparisons for 102 channels.

#### Early stage of active learning vs. passive listening (task effect).

First, we examined whether the task load by itself led to changes in ERFs evoked by the auditory pseudowords. To this end, we compared ERFs evoked by the same stimuli in the passive listening and active learning blocks. Since there were no movements during the passive block, for this comparison we used only those stimuli from the active learning block that did not trigger movements, i.e., the NPW pseudowords. We extracted the first seven trials for each of the four NPW pseudowords from the passive condition and an equal number of trials from the active learning condition, and calculated GFP over the whole sensor array. The GFP time courses were compared between the passive and active conditions using the paired two-tailed t-test with FDR correction for multiple comparisons for 960 time points within the stimulus-related epoch from −700–2500 ms.

#### Stimulus-locked ESL vs. ASL contrast (learning effect).

To test for possible enhancement and prolongation of stimulus-locked activity during the post-stimulus period at the advanced stage of learning, we compared the evoked magnetic field response between ESL and ASL conditions for both types of stimuli. Based on the “retrospective” hypothesis, we expected a stronger learning effect for newly learned “action” APW pseudowords compared to non-action-related NPW stimuli, assuming the learning effect should reflect the temporal integration of auditory and motor activation rather than just pseudoword memorization. If the learning effect were primarily driven by the retrieval of learned pseudowords and their movement or no-movement associates, both APW and NPW stimuli would be expected to show a similar increase in neural responses under the ASL condition.

First, for each stimulus type we calculated time courses of the GFP signal in the ESL and ASL conditions and compared them at each time point (with FDR-correction for a number of time points). As predicted, no significant learning effect was found for NPW pseudowords (see Results section), thereby, we examined further APW stimuli only. To evaluate scalp topography of the ESL-ASL differences, we chose an approximately 200-ms period during which the learning effect was significant at each consecutive time point, without discontinuity. For each sensor separately, we averaged RMS values within the chosen time window, and contrasted ESL and ASL conditions using the paired t-test with FDR corrections for multiple comparisons over 102 combined gradiometers.

Additionally, we plotted topographical maps for successive 100-ms time windows, both in APW and NPW trials before and after learning, as well as differential response to learning for each trial type (APW ESL vs APW ASL; NPW ESL vs NPW ASL).

#### Response-locked ESL vs. ASL contrast.

We tested whether the putative learning-induced increase in auditory cortex activation was present in response-locked ERFs. Only the APW trials, which involve motor responses, were considered. This analysis aligned MEG signals to the movement onset, while the information about the time point of word onset was jittered due to differences in reaction times.

First, for response-locked data, we compared GFP time courses for ESL and ASL conditions, using the two-tailed paired t-test, at each time point separately, with FDR correction for the number of timepoints in the whole epoch (750 time points within the response-related epoch from −1500 to +1000 ms).

Next, we attempted to evaluate the topography of the learning effect. In order to allow comparison with the stimulus-locked data, we chose a 200-ms time window. We selected the interval close to the motor response in order to capture processes directly related to the movement preparation, but we omitted 100 ms immediately preceding the response to avoid an onset of myographic activity related to button/pedal press [55]. The resulting time window from −300 to −100 ms before the response roughly corresponded to the interval that we defined as the most significant for stimulus-locked data (see above). For each combined gradiometer, RMS values were averaged across the time window of interest in each condition separately and then subjected to the paired t-test with FDR correction for multiple comparisons for 102 combined gradiometers.

In addition, we plotted the topographical maps of successive 100-ms time windows over the second before the movement onset before and after learning, as well as the differential response between the ESL and ASL stages of learning (ASL minus ESL).

One could argue that the observed learning effects in the response-locked data might be spurious, related to differences in response time between ESL and ASL conditions. Considering that the RTs were shorter in the ASL than in ESL condition, the increase in the response-locked activity in the ASL could be explained by the mere fact that response-locked ERFs became closer to the stimulus and, thus, were contaminated by stimulus-locked activity. To control for the difference in the response latency, we repeated the analysis for the data matched for the condition-averaged RT (the results of this analysis are presented in S1 Appendix in the Supporting Information).

#### Comparison between stimulus-locked and response-locked effects.

At the next step, we compared the strength of the learning effects in the stimulus-locked and response-locked data. This comparison was crucial for differentiating between the “retrospective” and “prospective” explanations of the enhanced evoked response at the advanced stage of learning, since the effect we observed in the movement-related data, could be simply a carry-over effect of the power increase in the stimulus-locked ERFs. We expected that, if the increase in the auditory cortex activation was indeed related to the movement preparation, the learning effect should be stronger in the response-locked data compared with the stimulus-locked.

With this goal in mind, we directly compared stimulus-locked and response-locked ERFs before and after learning, within the two time windows of equal length of 200 ms: the window of the most significant ESL-ASL differences in the stimulus-locked response (see above) and the chosen ‒300 – ‒100 ms window in the response-locked signal. For this purpose, we took only those combined gradiometers that were statistically significant in both analyses. The RMS values for these channels were averaged over respective 200-ms windows and subjected to repeated-measures ANOVA with two factors: learning stage (ESL and ASL) and ERF locking (stimulus-locked and response-locked). Post-hoc pairwise comparisons were made using the Tukey HSD test.

### Source-level analysis

#### MRI scanning, co-registration and source estimation.

Participants underwent MRI scanning with a 1.5T Philips Intera system for further reconstruction of the cortical surface. Individual structural MRIs were used to construct single-layer boundary-element models of cortical gray matter with a watershed segmentation algorithm (FreeSurfer 4.3 software; Martinos Center for Biomedical Imaging, Charlestown, MA, USA). Individually recorded head shapes were then co-registered to this mesh using fiducial points and around 60 individually digitized scalp-surface points. A grid spacing of 5 mm was used for dipole placement, which yielded 10,242 vertices per hemisphere.

Both magnetometer and planar gradiometer data were used to compute sources. The evoked data were downsampled to 100 samples per second. A noise-covariance matrix was computed for each subject from the empty-room data recorded immediately before the experiment. The noise-covariance matrix and the forward operator were combined into a linear inverse operator using sLoreta algorithm implemented in the MNE software suite using default parameters. The source estimates were morphed onto the standard MNI brain using the surface-based normalization procedure implemented in MNE [45]. The data were baseline-corrected at each vertex for the averaged current strength computed over the pre-stimulus −500 − −50 ms interval.

#### ESL vs ASL contrast.

We performed source reconstruction to allow for a more detailed description of the brain areas involved in the observed sensor-level effects of learning (ESL vs ASL differences) for both stimulus-locked and response-locked signals. Only trials with the motor responses were included in this analysis (i.e., APW trials).

First, for each time window of interest that was specified during the sensor-level analysis (for both stimulus-locked and response-locked activation, see above), we determined cortical areas involved in the neural response during ESL and ASL conditions separately. For each vertex, the cortical evoked current was averaged across the designated time windows at each condition and contrasted by means of the paired two-tailed t-test. To correct the results for multiple comparisons, we used the same FDR procedure as at the sensor level but across the entire set of vertices of the cortical surface instead of the MEG channels.

The cortical regions with the most reliable statistical effect were defined based on the Destrieux probabilistic atlas of the human brain [56] (see Results). Then, we extracted time courses of the source current from all significant vertices in each of the selected areas, under each condition separately. The resulting time series were averaged across the vertices for ESL and ASL conditions and, then, subjected to the point-by-point comparison by the paired two-tailed t-test, with FDR-correction for the number of time-points (250 time points corresponding to 2500-ms epoch).

#### Response-locked ERFs for movements made by each hand separately.

For the analyses described above, the epochs were selected in such a way that hand and foot movements were pooled together in equal proportion, thus obscuring any movement-related somatotopic effects in the sensory-motor cortex. To uncover activity related to movement preparation, we conducted an additional analysis specifically focusing on trials involving hand movements, distinguishing between those performed with the left and right hand. We extracted all correct APW trials involving hand movements from the active learning and active performance blocks separately. To improve the signal-to-noise ratio, we used all relevant epochs from each block (24.2 ± 11.7 in the active learning block, 35.9 ± 4.5 in the active performance block, mean ± SD). Then, we reconstructed time courses of cortical response in four regions of interest (see Results for details). Point-by-point comparisons between ESL and ASL conditions were made as described above.

## Results

### Behavioral performance

All participants successfully acquired the rules linking the eight different pseudoword cues with the movements of specific body extremities – or with withholding a movement – through active operant learning. The analyses below are reported for those 19 participants who had a sufficient number of trials in the active learning block and were included in the main dataset (see Methods). Across the active learning block, the mean proportion of correct responses was 0.71 (SD = 0.06), being significantly different from that of chance (t(18)=25.76, p < 0.001, Cohen’s d = 5.9). The correct response rate increased over the active learning block as learning proceeded. Fig 1D represents the cumulative record of correct responses as a function of trials in the active learning block in individual participants and at the group level. Inspection of the plots confirmed the general tendency for continuous improvement from an untrained, chance level of responding to a near perfect performance.

During the active performance block, all participants scored highly on the pseudoword-action association task (mean proportion of correct responses was as high as 0.97, SD = 0.04), indicating that they had successfully discovered and retained in memory the rules linking each of the eight auditory cues with the respective actions or non-action. As can be seen in Fig 1E, the participants maintained the near-ceiling performance level throughout the entire performance block, with the mean proportion of correct responses being well above 80%. There were no statistically significant differences in accuracy between APW and NPW trials both during the active learning block (70.8 ± 9.0 and 70.7 ± 6.7% for APW and NPW respectively, t(18)=0.06, p = 0.95) and during the active performance block (96.0 ± 3.5 and 97.3 ± 4.3% for APW and NPW respectively, t(18)=1.59, p = 0.13).

The RTs in the APW trials significantly shortened from the active learning block to the active performance block (1387 ± 171 vs. 1285 ± 114; t(18)=3.46; p = 0.003, Cohen’s *d* = 0.79). As expected, the decision to execute the cued motor response became easier as learning proceeded. Notably, LMEM analysis revealed that there was a significant negative relation between a successive trial number and RT in that trial within the active performance block (F(1, 2910.31)=4.31, p = 0.038) due to progressive shortening of motor response latency toward the end of this block. The factor of limb was also significant (F(3, 2910.02)=15.78, p < 0.001), with leg movements being significantly slower than hand movements. However, there was no interaction between the two factors (F(3, 2910.21)=2.36, p = 0.069) revealing that the tendency of shortening RTs throughout the active performance block was present for all extremities; this indicates that strengthening of auditory cue-motor association proceeded during the advanced learning stage at similar speed for all four movement types.

In addition, we compared mean RTs in the subset of trials chosen for the MEG analysis (see Methods for the details): the RTs taken from the beginning of the active learning block were significantly longer than the ones from end of the active performance block (1345 ± 105 ms vs 1264 ± 117 ms for APW ESL and APW ASL conditions respectively; t(18)=3.51, p = 0.002, Cohen’s d = 0.81).

### Stimulus-locked ERFs

#### Sensor-level analysis: early stage *of* active learning vs. passive listening (task effect).

**Fig 2:**
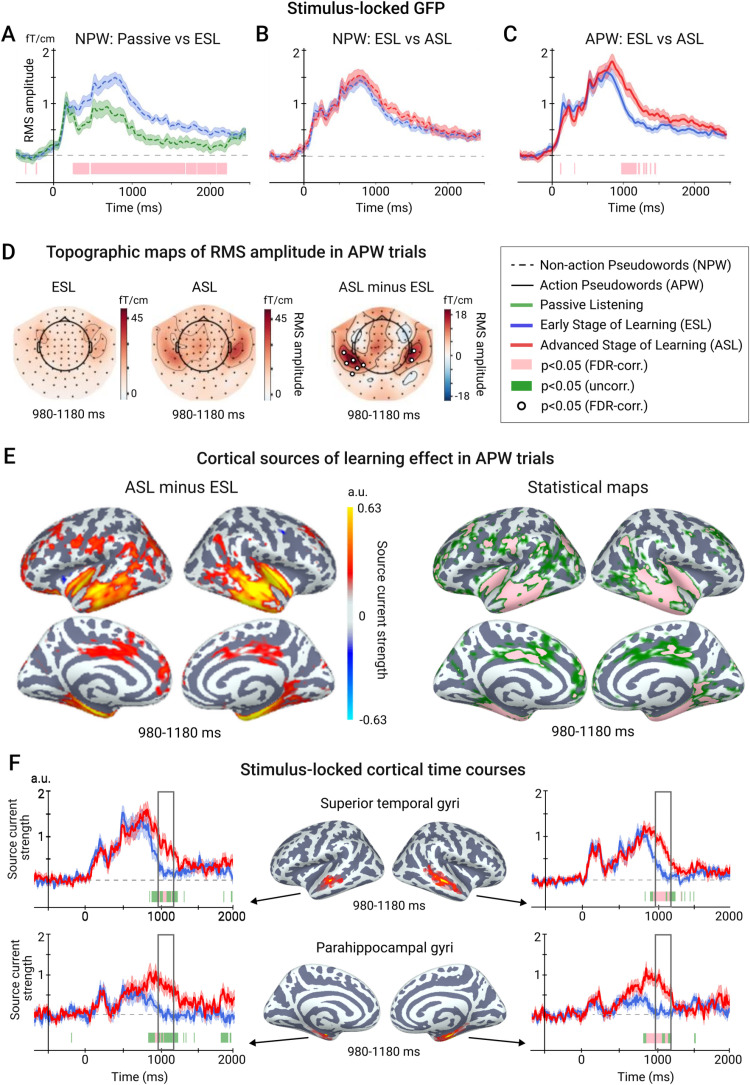
Learning effect in the stimulus-locked analysis. A. Time courses of global field power (GFP) in the first passive listening and early stage of learning (ESL) conditions in response to non-action related pseudowords (NPW). A vertical line corresponds to the stimulus onset. Here and hereafter, the shaded area around a time course represents the standard error of the mean (SEM). Colored horizontal bars under time courses represent time intervals with the significant between-condition differences (pink represents p < 0.05, FDR-corr.; green – p < 0.05, uncorr.). B. Time courses of GFP at early and advanced stages of learning (ESL and ASL) conditions in response to non-action related pseudowords (NPW). C. Time courses of GFP at ESL and ASL conditions in response to action related pseudowords (APW). D. Topographic maps of the mean RMS signal within the 980-1180 ms post-stimulus interval for APW stimuli during ESL and ASL conditions (left panels) and their difference (right panel). Here and hereafter, white circles indicate sensors with significant ESL-ASL differences (p < 0.05, FDR-corr.). The small head-shaped outline within each map indicates the orientation of the head inside the MEG helmet. E. Cortical sources of ESL-ASL differences in response to APW stimuli in the 980-1180 ms post-stimulus interval. The left panel depicts the difference in amplitudes of source currents between ESL and ASL conditions (thresholded by p < 0.05, uncorr.). The right panel demonstrates statistical maps of the between-condition differences: green color represents p < 0.05, uncorr.; pink color – p < 0.05, FDR-corr.). F. Time courses of source current strength from selected cortical regions. The cortical map in the middle displays the vertices with significant ESL-ASL differences in the 980–1190 ms interval within the selected region. The time courses represent an average across the significant vertices in the region.

As the first step, we contrasted the neural responses triggered by the pseudowords at the early stage of the learning to a control condition, when participants listened to the same auditory stimuli without any specific task in mind during the first passive block (Fig 1B). Since in the latter condition participants did not perform any movements, we included only NPW trials in this analysis. Point-by-point comparisons of the GFP time courses between the two conditions (Fig 2A) showed a highly significant task-related increase of the stimulus-locked response, which started at ~200 ms after the stimulus onset and sustained until at least 1500 ms.

The straightforward explanation of greater strength and duration of the auditory neural response at the ESL compared with the passive condition might be the increased attentional and memory load during the task performance. If the role of attention is to facilitate the acquisition and memorization of cue-outcome associations [57], it is plausible that the amount of attention paid to the auditory cues is greatest at the initial stage of learning. Following the same line of thinking, one could expect that the attentional boost effect on the neuromagnetic response should diminish with practice and task rule acquisition. In contrast, the associative learning hypotheses, as described in the Introduction, lead to the opposite prediction. Therefore, to separate the putative contribution of active learning from that of attention, we further compared stimulus-locked ERFs between the ESL and ASL conditions.

#### Sensor-level analysis: ESL vs. ASL (learning effect).

To assess whether auditory cue-evoked activity diminishes or increases over the course of learning, we performed point-to-point comparison of the GFP time courses between ESL and ASL for NPW and APW stimuli separately. As shown in Fig 2B, the GFP evoked by the NPW pseudowords did not differ between the ESL and ASL conditions at any time point (p > 0.05, FDR-corr.), whereas the neural response to the APW pseudowords (Fig 2C) increased significantly at the advanced stage of learning, approximately from ~980 to ~1180 ms after the stimulus onset (p < 0.05, FDR-corr.). Thus, the learning specifically enhanced the late stages of the stimulus-evoked response — but only for the APW stimuli associated with motor actions.

The topographical distribution of the learning-induced effect in the 980–1180 ms time window comprised two roughly symmetrical spatial clusters over left and right temporal regions (p < 0.05, FDR-corr.) (Fig 2D). For illustrative purposes, we also depicted successive snapshots of the ERF scalp topography throughout the period of the post-stimulus response in the ESL and ASL conditions (see S2 Appendix). As one can see in the RMS time courses and series of topographical plots, the learning effect persisted for a substantial time between the stimulus presentation and the response onset.

#### Source-level analysis: ESL vs. ASL (learning effect).

To assess the neural sources of the learning-induced effects in the APW trials that we had statistically demonstrated in sensor space, we performed the source localization analysis. The cortical sources were localized for the time window between 980 and 1180 ms post-stimulus onset, during which we observed the greatest learning effect at sensor level (Fig 2E). Consistent with the scalp topographies in Fig 2D, learning predominantly modulated the activity in the lateral and medial regions of the left and right temporal lobes, particularly the superior and inferior temporal sulci (STS and ITS, respectively), the parahippocampal gyri (PHG) and posterior insula (p < 0.05, FDR-corr.).

The time courses of the source current, averaged across the vertices with significant ESL-ASL difference in the STS and PHG regions (see Table 2 for MNI coordinates of the statistical maxima), were compared between the ESL and ASL conditions using paired t-tests (Fig 2F). The results aligned with those observed at the sensor level: the stimulus-related responses in the ESL and ASL conditions differed significantly between approximately 900 and 1200 ms post-stimulus onset, displaying similar dynamics bilaterally in both the STS and PHG regions (p < 0.05, FDR-corr.).

**Table 2:** Brain regions with the most reliable ESL-ASL differences in the stimulus- and response-locked data. MNI coordinates are given for the most significant vertices within the standard anatomical labels [56].

Brain region	MNI coordinates of the most significant vertex	
**Stimulus-locked analysis**	**Response-locked analysis**
Left parahippocampal gyrus	(−23.3, −12.7, −28.8)	(−20.5, −14.2, −26.8)
Right parahippocampal gyrus	(22.8, −20.3, −24.1)	(22.8, −20.3, −24.1)
Left superior temporal sulcus	(−51.9, −17.5, −8.0)	(−47.9, −24.9, −10.3)
Right superior temporal sulcus	(44.6, −28.9, −6.6)	(46.4, −25.6, −10.6)

In summary, both sensor- and source-level results indicated that learning of the stimulus-response associations slowed the decay of the stimulus-related neuromagnetic response during the interval between the stimulus and the cued action. This significant learning effect on stimulus-locked activation was observed only for the auditory stimuli that prompted a specific body movement, not for those requiring no movements. The cortical areas most affected by learning during the APW trials were in the lateral and medial temporal regions of both hemispheres. The learning-related modulations emerged after the peak of the auditory response and persisted until roughly the end of the delay period between the stimulus and movement onset.

### Response-locked ERFs

#### Sensor-level analysis: ESL vs. ASL (learning effect).

The next step of the analysis was to examine the learning effect on the response-locked activity. As the correct NPW trials did not contain overt motor responses, this part of the analysis was conducted only for APW pseudowords.

**Fig 3:**
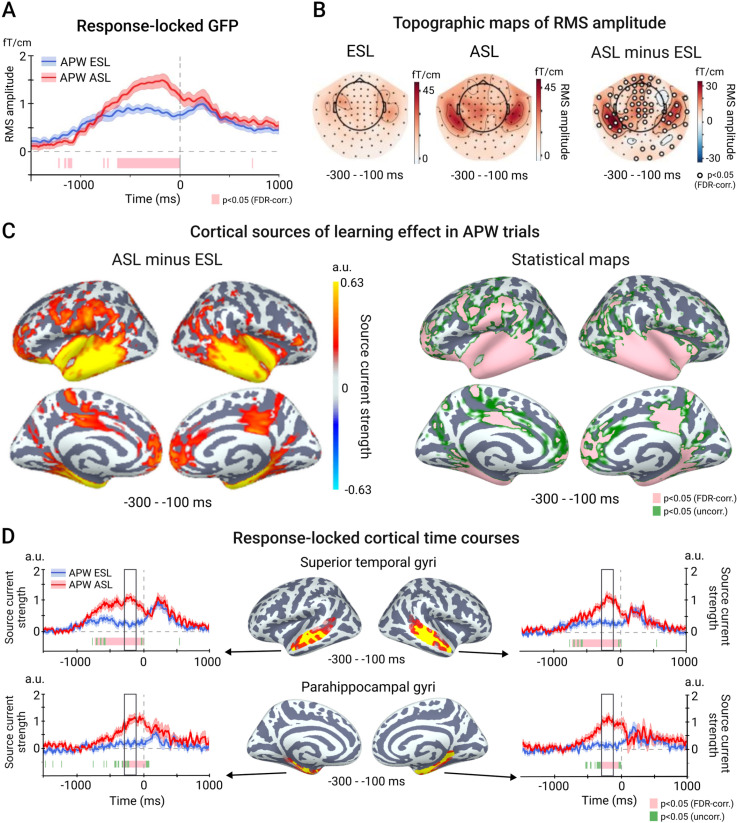
Learning effect in the response-locked analysis. A. Time courses of global field power (GFP) at early and advanced stages of learning (ESL and ASL) conditions during trials with action related pseudowords (APW). A vertical dashed line corresponds to a movement onset. B. Topographic maps of the mean RMS signal within the −300 – −100 ms pre-movement interval during ESL and ASL conditions (left panels) and their difference (right panel). C. Cortical sources of ESL-ASL differences in the −300 – −100 ms pre-movement interval. The left panel depicts the difference in amplitudes of source currents between ESL and ASL conditions (thresholded by p < 0.05, uncorr.). The right panel demonstrates statistical maps of the between-condition differences: green color represents p < 0.05, uncorr.; pink color – p < 0.05, FDR-corr.). D. Response-locked time courses of source current strength from selected cortical regions. The cortical map in the middle displays the vertices with significant ESL-ASL differences in the −300 – −100 ms pre-movement interval within the selected region. The time courses represent an average across the significant vertices in the region.

On the response-locked APW epochs, the t-test comparing point-by-point GFP amplitudes revealed a highly significant learning-related increase in the ASL compared with the ESL condition, which started about ~600 ms before movement onset and lasted until the movement onset itself (p < 0.05, FDR-corr.) (Fig 3A). Within this extended period of the significant learning effect, we selected a specific time window of interest—spanning from 300 to 100 ms before movement onset — to capture motor preparation. Fig 3B shows that the gradiometers exhibiting the most significant learning-induced changes were located bilaterally over the temporal lobes. Scalp topography maps at consecutive 100 ms time windows show that these statistical clusters persist throughout the entire pre-movement period (S2 Appendix).

The systematic reduction in RTs in the ASL condition, compared to the ESL condition, may have brought the pre-movement period closer in time to stimulus-driven processing, potentially causing spurious ESL-ASL differences in the response-locked data. To ensure that the observed effect was not a technical result of RT difference, we selected subsets of trials in such a way that the group mean RTs did not differ between ESL and ASL conditions (mean RT ± SD: 1263 ± 119 ms vs 1256 ± 123 ms; t(18)=1.18; p = 0.25). The learning effect remained significant in the RT-matched data for approximately the same protracted period of time before the movement onset (approximately from −500–0 ms) as in the original data set (p < 0.05, FDR-corr.) (S1 Appendix).

#### Source-level analysis, ESL vs. ASL (learning effect).

The cortical areas underlying the learning effect on the response-locked ERFs in the time window of interest (−300 – −100 ms) are shown in Fig 3C. The set of cortical regions underlying the greater response-locked ERF in ASL versus ESL conditions closely resembled that observed for the stimulus-locked response, though it was more extensive. Specifically, learning enhanced the response-locked activation in widespread areas of the temporal lobe (including the STS, ITS, and PHG), the frontal pole, the posterior region of the mid-cingulate cortex, and the left inferior parietal cortex. The reconstructed time courses of the cortical activity from the STS and PHG areas (Fig 3D; see Table 2 for MNI coordinates of the most significant vertices) revealed that the significant learning-related differential activation (p < 0.05, FDR-corr.) started at the left STS ~ 700 ms before the movement onset, then, at around 450 ms, it became apparent in the right STS and in the PHG bilaterally. Once emerged, the learning effects persisted until the onset of the movement response, peaking approximately 200 ms before the movement execution.

Overall, our response-locked data showed that the learning-induced increase in temporal lobe activity was present not only in response to the auditory stimulus but also in anticipation of the upcoming motor response. To distinguish between the “retrospective” and “prospective” accounts of this increase, a direct comparison of stimulus- and response-locked activity was necessary.

#### Learning effect in response-locked vs. stimulus-locked data.

**Fig 4:**
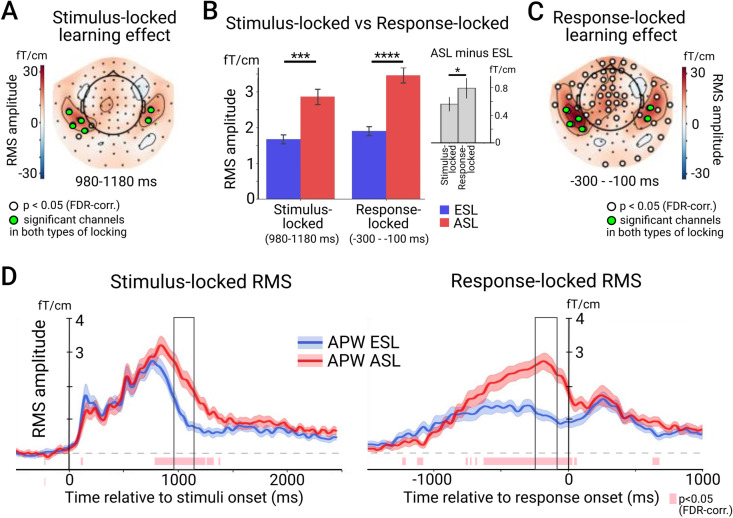
Comparison of the strength of the learning effect between the stimulus-locked vs. response-locked data. A. The topographical map of differential RMS amplitude between ESL and ASL in the stimulus-locked data during the time interval of interest. Here and elsewhere, white circles indicate channels with significant ESL-ASL differences (p < 0.05, FDR-corr.), green circles mark the subset of channels which were significant in both analyses. B. Bar graphs represent mean values of RMS over the selected subset of channels at time of interest during ESL and ASL conditions in stimulus- and response-locked analyses. On the insert, a bar graph shows the difference in RMS amplitudes between ESL and ASL conditions in the stimulus- and response locked data. Whiskers indicate 95% confidence intervals. Asterisks denote the level of significance: * < 0.05, **p < 0.01, ***p < 0.001, ****p < 0.0001. C. The topographical map of differential RMS amplitude between ESL and ASL in the response-locked data during the time interval of interest. D. Time courses of RMS calculated over the subset of channels which displayed significant ESL-ASL differences in both stimulus- and response-locked analyses. The time course on the left is aligned to the stimulus onset, the time course on the right – to the onset of the motor response. The black rectangles indicate the time intervals of interest.

To contrast the learning-induced effects in the stimulus- and response-locked neural responses, we compared the response-locked data averaged across the pre-movement period of −300 – −100 ms before the response onset with the stimulus-locked data averaged across the interval of the most reliable ESL-ASL difference in the stimulus-locked analysis (980–1180 ms after the stimulus onset). Considering the mean RT of ~1300 ms, two temporal intervals roughly overlapped. The spatial clusters of gradiometers showing significant effects in the ASL vs ESL contrast (p < 0.05, FDR-corr.) had similar maxima for both types of locking (Fig 4A, C). However, the learning effect in the response-locked RMS was notably more extensive, involving gradiometers covering bilateral temporal and midline frontal regions.

To compare the stimulus-locked and response-locked data, we focused on those sensors that were significant in both analyses; they overlay the left and right temporal cortex. Point-by-point comparisons of the RMS time courses, representing the average across this subset of temporal gradiometers, showed a significant increase in the neural response in the ASL condition compared to the ESL condition for both stimulus- and response-locked activity within the protracted interval preceding movement onset (Fig 4D).

To check whether the observed activation of the auditory-related temporal cortex prior to the movement onset results from the sustained stimulus-locked activation “leaking” into the response-locked neural responses, we directly compared the strength of the learning effects between the stimulus-locked and response-locked ERFs in this subset of common temporal sensors. The RMS values were averaged across the selected sensors and 200-ms windows of interest in the stimulus-locked and response-locked ERFs separately and subjected to ANOVA with the learning stage (ESL vs. ASL) and the type of locking (stimulus-vs. response-locked ERFs) as repeated measure factors. Both main factors were significant (learning stage: F(1,18)=31.89, p < 0.001, partial η^2^ = 0.64; locking: F(1,18)=16.12, p = 0.001, partial η^2^ = 0.47), as well as their interaction (F(1,18)=7.06, p = 0.016, partial η^2^ = 0.28). The learning-related amplitude increase was significantly greater in the response-locked compared with the stimulus-locked activity (t(18)=−2.72, p = 0.014) (Fig 4B). The larger learning effect in the response-locked ERFs than in the stimulus-locked ERFs in the temporal sensors refuted the possibility that the response-locked effect solely resulted from a spillover of the late parts of the stimulus-locked activity. To the contrary, the data do not exclude the possibility of leakage in the reverse direction—from the response-locked to the stimulus-locked activity. These findings suggested the presence of a distinct mechanism for the auditory cortex reactivation related to the movement preparation, consistent with the “prospective” hypothesis.

#### Learning effect for hand movements.

A key prediction of the “prospective” hypothesis is that the acquired association between the auditory cue and the movement of a specific body part should lead to a temporal coincidence of motor and auditory activation within the involved cortical areas. Yet, in the analyses reported above, the response-locked signal was averaged across four different body parts, and a respective somatotopic motor activation was blurred. Therefore, to examine the predicted co-occurrence in activation of auditory cortex and motor representation of the specific body part triggered by the readiness to initiate its movement, we focused on a well-studied MEG/EEG component called motor readiness potential, or motor readiness field (MRF) in MEG studies. The MRF is a contralaterally dominant slow field shift preceding unilateral movements that is considered as the MEG signature of motor preparation [58–60]. Cortical sources of the MRF are localized to the hand area in the primary motor and premotor cortex predominantly in the contralateral hemisphere [58]. Based on the notion of lateralized MRF as a strictly motor preparation component, we wanted to ensure that this MEG hallmark of motor activation was overlapping in time with the augmented auditory cortex activation preceding the hand movements.

**Fig 5:**
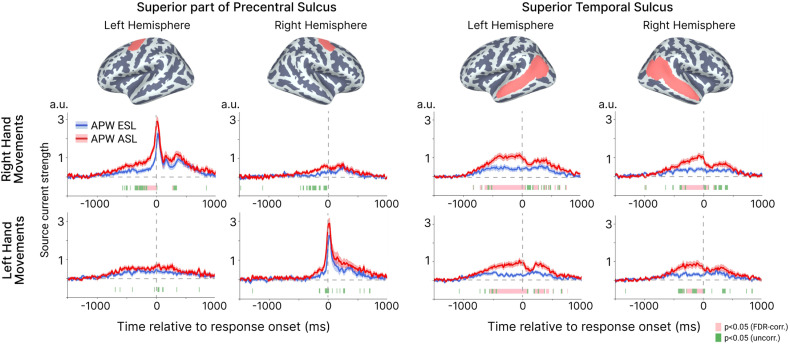
Learning effect during movements of the right and left hands. Each row represents response-locked time courses of source current strength from the superior part of precentral sulcus and superior temporal sulcus during right hand (upper row) and left hand movements (bottom row). The corresponding cortical regions are depicted on the lateral surface of both hemispheres. The time courses represent the average across the vertices in each region. A vertical dashed line indicates the movement onset. Colored horizontal bars under time courses represent time intervals with the significant between-condition differences (pink represents p < 0.05, FDR-corr.; green – p < 0.05, uncorr.).

To this end, we extracted response-locked signals for the right and left hand movements in both ESL and ASL conditions, and reconstructed time courses of average source current for the four cortical regions selected from the Destrieux atlas [56]. The first two regions comprised the superior part of the precentral sulcus from left and right hemispheres (Fig 5). These areas contain lateral premotor hand area [61], known to contribute to generation of the hand-related MRF [59]. The two other cortical regions included the whole STS label from the Destrieux atlas, chosen because of their association with lexical-phonetic aspects of speech processing, as demonstrated in fMRI and MEG studies [62–64]. As MEG has low sensitivity to radially oriented sources in the cortical gyri [65], the regions of interest were limited by the sulci surface.

Fig 5 shows the resulting time courses of cortical activity in the selected cortical regions during the right and left hand movements under the ESL and ASL conditions. Consistent with previous literature [58,66], we observed a bilateral slow amplitude shift (motor readiness activity) starting approximately 500 ms before movement. This shift was more pronounced for right-hand movements and eventually developed into a sharp increase in activity in the contralateral premotor hand area, peaking shortly after movement onset. The learning-induced STS activation also began around 500 ms before movement and persisted until the motor response was executed, with its onset and duration largely overlapping with that in the contralateral pre-motor hand area.

Thus, the enhancing effect of associative learning on the response-locked bilateral activation in the auditory cortex coincides temporally with the motor preparation processes specific to the referential movement.

## Discussion

The current study aimed to examine whether associative learning enhances temporal cortex activation during the time interval between the auditory pseudoword and the motor response. We indeed found such an increase both in stimulus-locked and response-locked neural responses in both lateral (including speech-related areas) and medial temporal cortices, although this increase was significantly more prominent in the response-locked data compared with the stimulus-locked data.

Comparing the neuromagnetic response at the early stage of learning to passive listening of the same pseudowords, we found that the requirement to memorize pseudoword-action associations enhanced and prolonged the stimulus-locked brain activity that peaked around 800 ms after pseudoword onset (Fig 2A). In our study, the word-form uniqueness point of the spoken pseudowords corresponded to ~360 ms after their onset, being the first moment when acoustic information required for pseudoword identification became available. Given this, the latency of the peak activity we observed agrees with the N400-like activity described for spoken word/pseudoword processing, which typically peaks at around 400 ms after the recognition point [67]. The relatively late timing of the N400 component also aligns with previous research on associative auditory pseudoword learning [68]. The observed augmentation of the stimulus-locked activity under the learning task is also in accord with previous studies that showed enhancement of the N400 component when attention is drawn to verbal stimuli, as opposed to its unattended processing [69,70]. Furthermore, the N400 component with an unusually long descending slope has been observed in lexical decision tasks where, as in our study, a pseudoword representation was actively maintained in short-term memory to guide selection of an appropriate overt verbal response [67].

Therefore, the lengthened N400m in our study likely results from the increased demands on attention and memory during the early learning stage, compared to passive listening.

This interpretation, however, does not explain the further increase in the stimulus-evoked neural responses observed over the course of learning (Fig 2C, D). Both focused attention and memory retention were likely required throughout the entire period of mastering the pseudoword-action associations. Yet, as learning progresses and stimulus-to-movement matching becomes more automatic, the load on memory and attention typically decreases [71]. This decrease should, in turn, reduce neural activation related to these cognitive processes. Therefore, it is unlikely that these general-domain processes alone account for the slowed decay of sustained stimulus-locked neural activation we observed at the advanced stage of learning—when errors had nearly disappeared and reaction times had significantly decreased, clearly indicating a high degree of automatization of the cued responses.

In contrast, this finding appears to agree with the “retrospective” associative hypothesis, which posits that during learning, the activation of an input representation should be extended to overlap temporally with programming of the motor output. Accordingly, we observed that the enhanced N400m response to pseudowords was sustained until the onset of the corresponding movement, particularly at the late stage of learning when the unique associations between each pseudoword and its corresponding body part movement had been reliably established (Fig 2C). In other words, this sustained activation emerged only when confidence in the accuracy of the association was high, and the auditory pseudoword cue unambiguously predicted the execution of the corresponding action. Moreover, since no differences were observed between the early and late stages of learning for non-action-related pseudowords (Fig 2B), we infer that the learning-induced changes in the N400m wave were specific to pseudowords requiring the selection, preparation, and execution of movements.

However, the learning-induced enhancement of temporal cortex activation was also evident in the response-locked data, which could support the “prospective” hypothesis. As with the stimulus-locked data, this enhancement became prominent at the advanced learning stage, when movements were strictly determined by the preceding pseudoword, spanning a similar time interval and exhibiting a similar cortical topography in both data types (cf. Fig 4A, C). Notably, the learning effect was more pronounced in the response-locked than in the stimulus-locked response (Fig 4B). These suggest that the stronger response-locked activation, induced by associative learning, might leak into the late stages of stimulus-triggered activity, potentially inflating or even creating the observed learning effect in the stimulus-locked response. While this does not entirely rule out the “retrospective” hypothesis, it lends stronger support to the “prospective” explanation.

The learning-induced increase in the response-locked neural activity before the movement onset originated predominantly from medial and lateral temporal cortices in both hemispheres (Fig 3C). Parahippocampal gyrus on the medial surface with direct connections to the hippocampus is considered as a part of hippocampal memory circuits [72] and has been previously implicated in establishing associations between diverse elements [73]. It is generally agreed that the hippocampal system is a convergence zone that receives signals from all the modalities and supports binding diverse representations into associative memories [74,75]. One of the possible approaches sees the hippocampus as an “indexing system” which points to the distributed neocortical regions that store memorized representations [76,77]. We speculate that the enhancement of parahippocampal gyrus activity at the advanced stage of learning reflects the process by which pairs of simultaneously activated representations of the acoustic pseudowords and corresponding motor programs become jointly indexed and associated.

On the lateral surface of the temporal lobe, regions contributing to the learning effect on movement-evoked neural response to action pseudowords included the superior and inferior temporal sulci, known to be involved in the phonological and lexico-semantic processing of real words [41,64,78]. This involvement suggests that, as pseudowords were gradually acquiring the meaning of action words, speech-processing resources became actively engaged during movement preparation, up until just before movement onset. The learning-related effect was also strongly pronounced in extrahippocampal neocortical structures implicated in semantic memory, particularly the anterior temporal lobe (ATL) and the anterior parahippocampal regions (Fig 3C). The ATL, in particular, is hypothesized in clinical research and computational studies to function as a hub for integrating multimodal information into more abstract semantic knowledge [15,79,80], and may be critical in supporting novel semantic associations like those introduced in our study.

A crucial prediction of the “prospective” hypotheses was a coincidence in time of the reactivation of the acoustic stimulus representation and preparation of the motor response. However, because the previous analyses averaged data across movements of all four extremities in equal proportion, any somatotopically specific motor activation was obscured. To reveal somatotopic activation in movement-locked responses, we considered trials with right and left hand movements separately and indeed observed that movement-locked augmentation of the temporal cortex activity co-occurred with the lateralized motor readiness potential in the contralateral pre-motor hand area – a signature of movement programming [60,66] (Fig 5). Thus, this finding further supports the prospective hypotheses that predicted simultaneous reactivation of the memorized input representation and activation of the motor response program.

The protracted, gradually increasing activation that we observed in the temporal cortex before an upcoming movement resembles the slow rising waves that occur in anticipation of predictable auditory stimuli [81–84]. Such sustained “anticipatory” deflections belong to a broad class of slow event-related preparatory potentials (including stimulus preceding negativity, contingent negative variation, motor readiness potential and others), which are typically observed prior to predictable events. They are thought to reflect pre-activation of a specific representation which serves as a forward model of expected input or output [84,85]. In our task, sustained activation of the speech-related areas of the temporal cortex was triggered by the anticipation of movement associated with a specific auditory pseudoword, rather than by the auditory pseudoword itself. This suggests that at the advanced stage of learning, once the internal model linking acoustic pseudowords to the certain action is established, both the representation of word-form and its motor counterpart are activated during movement preparation. Our finding suggests the intriguing possibility that sustained anticipatory pre-activation may not be limited to cortical areas of the same modality as the predicted event but may also involve cortices that encode associations related to that event.

In this respect, the learning-induced reactivation of the auditory cortex before the upcoming movement reported here may be akin to a phenomenon of auditory-motor resonance – the bidirectional auditory-to-motor mapping that results in activation of motor cortical regions during listening to sounds produced by actions [86] and in activation of auditory cortices while performing actions (i.e., as playing a familiar melody on a mute piano keyboard [87]) (for extensive reviews, see [88,89]). It has been shown that even short-term learning can lead to action-triggered auditory neural activity. For example, after short trumpet rehearsal, the mute trumpet-based task involving only finger movements increased activation of the primary auditory cortex in non-musicians [90]. A novel finding in the current study, however, was that as a result of newly acquired associations between sound and action, the auditory activity occurred in anticipation of the participant’s own movement, rather than being triggered by the movement onset. This finding demonstrates that during the planning of upcoming actions in an active operant conditioning procedure, the brain reactivates representations of auditory cues in the auditory cortex, along with their associated motor counterparts.

There are several important limitations to this study. Future research should investigate the effects of artificially prolonged intervals between auditory pseudowords and referential actions to confirm whether, once association rules are established, action preparation alone can trigger auditory-related activation, even after the stimulus-locked temporal activity has faded. Furthermore, our findings are restricted to trial-and-error association learning with a strong implicit component [91]. Complementary cognitive learning strategies such as explicit encoding or fast mapping may involve distinct mechanisms [79,92], but see [93]. Beyond that, further investigation is needed to explore the retention of pseudoword memory traces acquired through operant conditioning, including whether sleep-related consolidation is necessary for long-term storage.

Taken together, our findings show that, as a result of the acquisition of one-to-one associations between auditory pseudowords and specific actions through trial-and-error search, the sustained temporal cortex activation is initially triggered by the presented pseudoword, and then re-occurs synchronously with initiation and preparation of the correct movement. This provides the first evidence that the adult brain, in order to form a novel association, relies on a “prospective” mechanism, which enables it to fill even a prolonged temporal gap between an auditory word and associated action. Such reactivation may enable binding via Hebbian plasticity, ultimately resulting in long-lasting synaptic changes within temporally correlated neural assemblies.

## Supporting information

S1 Appendix Response-locked ESL vs. ASL contrast: analysis on trials matched for the RT. (DOCX)

S2 Appendix Topographic maps of stimulus-locked and response-locked RMS signal. (DOCX)
