# Supplementary material for: Newly acquired word-action associations trigger auditory cortex activation during movement preparation: Implications for Hebbian plasticity in action word learning
Source: PLoS One. 2025 Jul 2;20(7):e0325977. doi: 10.1371/journal.pone.0325977 (PMC12221086; doi:10.1371/journal.pone.0325977)
Supplement: S1 Appendix — (DOCX) [file pone.0325977.s001.docx]

**S1 Appendix.** Response-locked ESL vs. ASL contrast: analysis on trials matched for the RT

The effects that we observed in the response-locked data and interpreted as the effect of learning during the interval of motor preparation could in fact be spurious, related to differences in response time between ESL and ASL conditions. Considering that the RTs were shorter in the ASL than in the ESL condition, the response-locked activity of interest could become closer to the stimulus and thus explain the observed increase in the ASL condition. To control for the difference in the response latencies, we repeated the GFP analysis on the subset of trials that did not differ in the average RT between ESL and ASL conditions (i.e. only relatively faster responses from the ESL condition and relatively slower responses from the ASL).

To achieve this, we used the following response time matching procedure in each subject. For each trial from the ESL condition, we attempted to select a matching trial from the ASL: a matched pair was assigned on condition that the difference in reaction times within a pair of selected trials was less than 150 ms. The trials were picked one by one in the order in which they were presented to the subject. At each step, matched pairs of trials were removed from the further search. After this procedure was completed, only matched trials were included in the further analysis, while unmatched trials were discarded. This procedure resulted in average 21.16 ± 2.58 trials per subject. The RTs in those selected trials were averaged in the ESL and ASL conditions and, then, subjected to two-tailed paired t-test. After assuring that the RTs in the subset of trials did not differ between ESL and ASL (mean RT ± SD: 1263 ± 119 ms vs. 1256 ±123 ms; t(18)=1.18; p=0.25), we repeated comparisons of time courses of GFP as described in Methods. As can be seen in S1 Fig. 1, the learning effect remained significant in the RT-matched subset of trials (both in stimulus- and response-locked analyses), supporting the notion that the observed increase in the movement-locked ERF was induced by learning rather than was caused by disparity in the RTs.


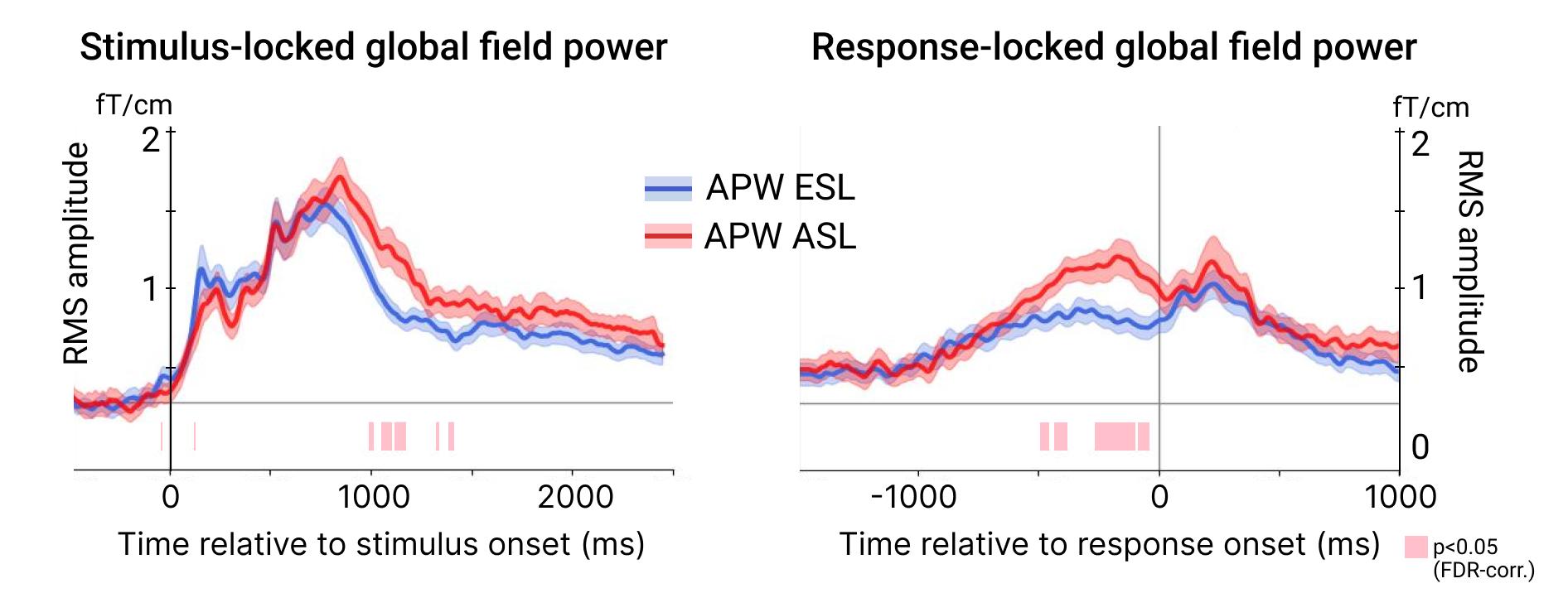


S1 Fig.1. Learning effect in trials matched for the response times. The time courses of the global field power at ESL and ASL on the left are aligned to the stimulus onset, the time courses on the right - to the onset of the motor response. The shaded area around a time course represents the standard error of the mean (SEM). The pink horizontal bars under time courses represent time intervals with significant between-condition differences (p<.05, FDR-corr.).
