# Supplementary material for: Newly acquired word-action associations trigger auditory cortex activation during movement preparation: Implications for Hebbian plasticity in action word learning
Source: PLoS One. 2025 Jul 2;20(7):e0325977. doi: 10.1371/journal.pone.0325977 (PMC12221086; doi:10.1371/journal.pone.0325977)
Supplement: S2 Appendix — (DOCX) [file pone.0325977.s002.docx]

**S2 Appendix.** Topographic maps of stimulus-locked and response-locked RMS signal


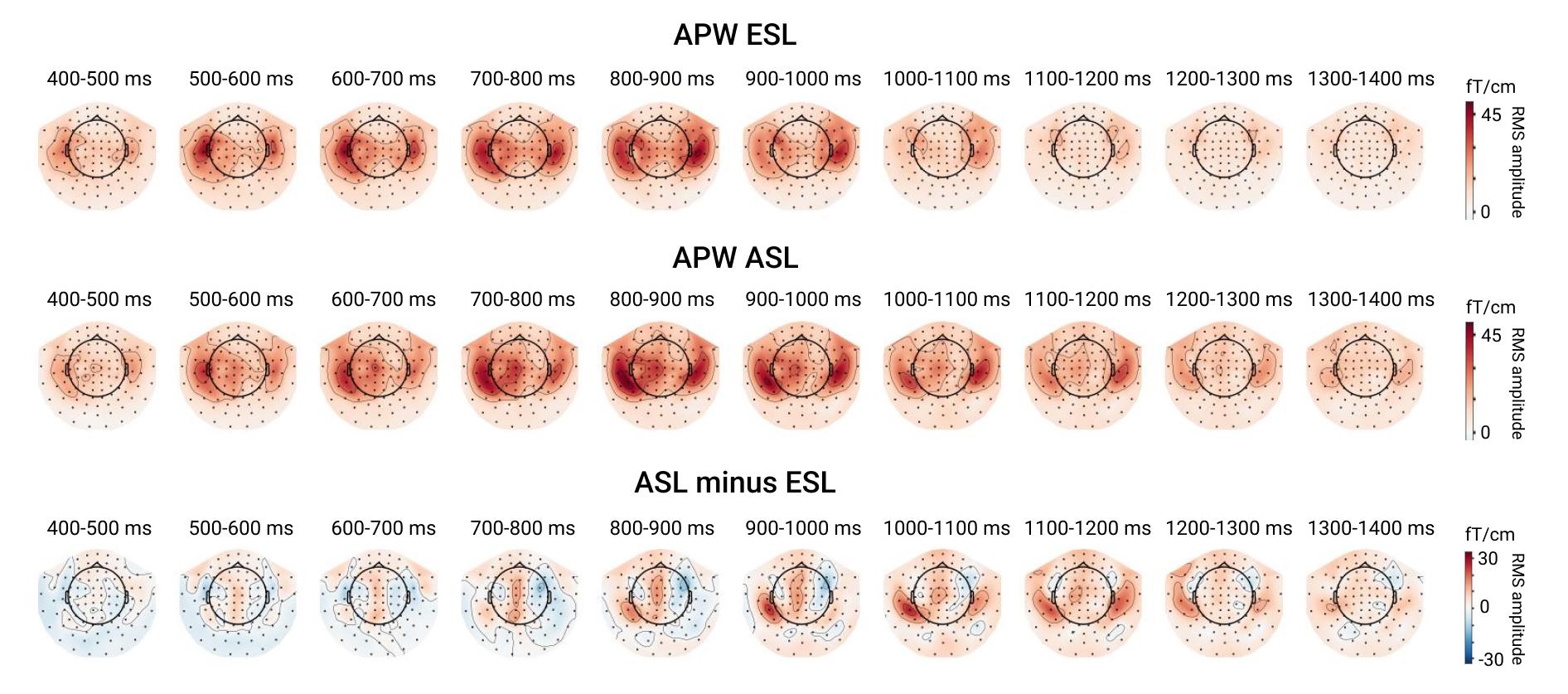


S2 Fig.1 Topographic maps of RMS signal at consecutive 100 ms time windows relative to the stimulus onset (t=0 ms). The upper row represents ERF topography in response to action-related pseudowords (APW) at the early stage of learning (ESL). The middle row represents the response to APW at the advanced stage of learning (ASL). The bottom row - the difference between ESL and ASL. Note the different scale for the differential activity.


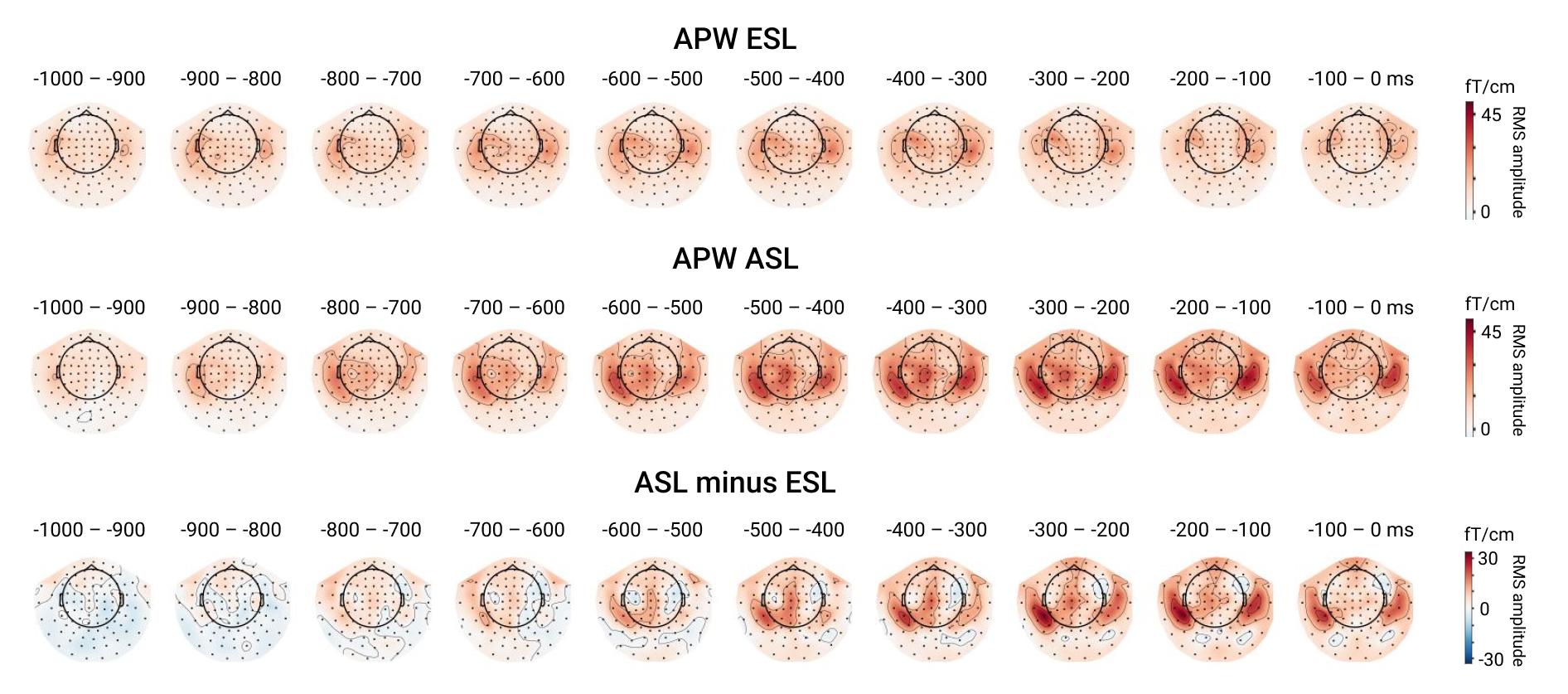


S2 Fig.2. Topographic maps of RMS signal at consecutive 100 ms time windows relative to the movement onset (t=0 ms). The upper row represents ERF topography in response to action-related pseudowords (APW) at the early stage of learning (ESL). The middle row represents the response to APW at the advanced stage of learning (ASL). The bottom row - the difference between ESL and ASL. Note the different scale for the differential activity.
